# Non-Negative matrix factorization combined with kernel regression for the prediction of adverse drug reaction profiles

**DOI:** 10.1093/bioadv/vbae009

**Published:** 2024-01-23

**Authors:** Yezhao Zhong, Cathal Seoighe, Haixuan Yang

**Affiliations:** School of Mathematical & Statistical Sciences, University of Galway, Galway H91 TK33, Ireland; School of Mathematical & Statistical Sciences, University of Galway, Galway H91 TK33, Ireland; School of Mathematical & Statistical Sciences, University of Galway, Galway H91 TK33, Ireland

## Abstract

**Motivation:**

Post-market unexpected Adverse Drug Reactions (ADRs) are associated with significant costs, in both financial burden and human health. Due to the high cost and time required to run clinical trials, there is significant interest in accurate computational methods that can aid in the prediction of ADRs for new drugs. As a machine learning task, ADR prediction is made more challenging due to a high degree of class imbalance and existing methods do not successfully balance the requirement to detect the minority cases (true positives for ADR), as measured by the Area Under the Precision-Recall (AUPR) curve with the ability to separate true positives from true negatives [as measured by the Area Under the Receiver Operating Characteristic (AUROC) curve]. Surprisingly, the performance of most existing methods is worse than a naïve method that attributes ADRs to drugs according to the frequency with which the ADR has been observed over all other drugs. The existing advanced methods applied do not lead to substantial gains in predictive performance.

**Results:**

We designed a rigorous evaluation to provide an unbiased estimate of the performance of ADR prediction methods: Nested Cross-Validation and a hold-out set were adopted. Among the existing methods, Kernel Regression (KR) performed best in AUPR but had a disadvantage in AUROC, relative to other methods, including the naïve method. We proposed a novel method that combines non-negative matrix factorization with kernel regression, called VKR. This novel approach matched or exceeded the performance of existing methods, overcoming the weakness of the existing methods.

**Availability:**

Code and data are available on https://github.com/YezhaoZhong/VKR.

## 1 Introduction

Adverse drug reactions (ADRs) affect human health and can lead to death in severe cases. Up to^ 2022^, 23 663 780 cases of ADRs have been reported, including 13 219 628 serious reports and 2 240 339 deaths (FDA 2022). There is a clear need to mitigate the impact of ADRs on human health and to avoid the costs of recalling approved drugs; however, research on ADRs is time-consuming and challenging. Therefore, there is significant interest in the development of accurate computational methods to predict ADRs. Progress has been reported towards this objective, particularly for the prediction of novel ADRs for existing drugs and for the prediction of drug–drug interactions (Sachdev and Gupta 2020), but methods for new drugs—the ADR profile prediction problem, still have great potential for development.

In recent years, various methods were developed for the ADR profile prediction problem. The first method Pauwels *et al.* (2011) used chemical substructure to predict ADRs, adopting Ordinary Canonical Correlation Analysis (OCCA) as well as sparse CCA (SCCA). As the ADR prediction studies develop, more features are being introduced to make predictions, and features are integrated by various strategies. For example, drug–drug interactions, chemical structures, single-nucleotide polymorphisms, and drug–target data have been adopted (Seo *et al.* 2020). In an extended version of kernel regression (KR), Yamanishi *et al.* (2012) proposed a Multiple Kernel Regression (MKR) method to integrate chemical structure data and protein target data of drugs. Zhang *et al.* (2016) and Zhang *et al.* (2017) developed a linear neighbourhood similarity method (LNSM) that uses a single feature to predict ADRs. Moreover, they developed two extensions, the LNSM–similarity matrix integration (LNSM–SMI) method and the LNSM–cost minimization integration (LNSM–CMI) method to integrate multiple features. There has also been a great deal of attention given to support vector machine (SVM) methods, which are among the most commonly used and mentioned methods in ADR prediction (Pauwels *et al.* 2011, Liu *et al.* 2012, Mizutani *et al.* 2012, Huang *et al.* 2013, Shaked 2016, Jiang *et al.* 2020). Even though some of the classical methods, OCCA, SCCA, logistic regression (LR), naïve Bayesian (NB), K-Nearest Neighbour (KNN), and Random Forest (RF), have been shown to be inferior to SVM, researchers have revitalized them with extra strategies (Pérez-Nueno *et al.* 2015, Lee *et al.* 2017, Seo *et al.* 2020).

The data imbalance in drug–ADR data necessitates the careful selection of appropriate metrics since the number of negative cases far exceeds the number of positive cases. The presence of imbalance makes it difficult for methods to learn the patterns present in the minority class. Additionally, when evaluation metrics are not adjusted to account for the class imbalance, they may mask this difficulty by excessively emphasizing the number of true-negative (TN) cases. However, it is also essential for methods to reach a high number of TN cases, since we need to exclude the ADRs that do not occur for a drug in order to make useful predictions. Thus, the main aim of this study was to develop a method that performs well under the appropriate metrics in the context of severely imbalanced data. The area Under the Precision-Recall Curve (AUPR) and the Area Under the Receiver Operating Characteristic Curve (AUROC) were used to measure how well the methods predict the minority class and how well the methods discriminate the positive and negative cases.

Notably, none of the studies mentioned above considered the importance of comparing performance against the most naïve method—using the mean values as the prediction scores for each ADR. Under this method, each drug is predicted to have a given ADR according to the proportion of all drugs that have the ADR. Unexpectedly, the naïve method outperformed most existing methods, as measured by AUROC, at little computational cost. Therefore, we considered the naïve method as an important baseline in our study.

In this study, we found that the existing methods for ADR profile prediction might not be rigorously evaluated. So a comprehensive comparison was carried out to evaluate different kinds of existing methods, including the naïve method. We used 5×4 Nested Cross-Validation (5×4 Nested CV) to avoid performance overestimation arising from hyperparameter tuning. As an additional precaution, we set aside a hold-out set (test set) in the early stage of the project and avoided accessing it until the very late stage. Surprisingly, the naïve method we designed as a baseline outran most of the existing methods. Among all the methods we reviewed, KR had the best performance in AUPR. Although it reached the best AUPR, it was mediocre under AUROC. Nevertheless, KR is still a potential method to be improved. We considered non-negative matrix factorization (NMF) could help to reduce the noise in the imbalanced ADR data since it is a well-studied dimension reduction method; therefore, we proposed a hybrid method that combines NMF with KR called Kernel Regression on *V* (VKR). We adopted SIDER 4.0 as the ADR data and included data from the Drug–Gene Interaction Data Base (DGIdb) as a novel feature, which has not previously been used for ADR profile prediction. In addition, we also chose the most commonly used data—chemical structure fingerprints (Pauwels *et al.* 2011, Liu *et al.* 2012, Yamanishi *et al.* 2012), as input feature data. We found that VKR outperformed other existing methods using both single features and integrated features, without sacrificing the ability to predict negative cases of ADRs.

## 2 Methods

### 2.1 Data sets

#### 2.1.1 Drug–ADR pairs data

We used the SIDER 4.1 database (Kuhn *et al.* 2010, 2016). SIDER 4.1 is an ADR dataset containing drug–ADR data, which provides labels for our ADR predictions. One thousand four hundred and thirty drugs and 5868 ADRs are included, and there are 139 756 drug–ADR pairs in total. We searched features from DGIdb and PubChem for these drugs. SIDER 4.1 was downloaded on 12 April 2021.

#### 2.1.2 Drug–gene interaction database

We retrieved drug–gene interaction (DGI) pairs from DGIdb 4.0 (Freshour *et al.* 2021). We mapped the drug names from SIDER 4.1 to DGIdb 4.0 and found 937 drugs in common. DGI pairs (11 378) were filtered from the common drugs corresponding to 2050 genes. DGIdb 4.0 was downloaded on 20 April 2021.

#### 2.1.3 Drug chemical structure fingerprints

We retrieved the drug chemical structure fingerprints (Chem) by mapping the CIDs of 1430 drugs from SIDER 4.1 to PubChem. PubChem fingerprints are binary vectors that can represent the chemical structures of drugs (Kim *et al.* 2023). Eight hundred and seventy-five of 1430 drugs were found to have chemical fingerprints in PubChem. We used the ChemmineR (Cao *et al.* 2008) and ChemmineOB (Horan and Girke 2022) packages in R to generate the fingerprints. We used the fingerprint type FP2, which is a path-based fingerprint indexing molecular fragments. These data are the most commonly and widely used features for ADR profile prediction. The Chem dataset was downloaded on 16 December 2021.

#### 2.1.4 Dataset splitting

If a drug has neither DGI nor Chem features, we cannot make predictions on the new drug ADRs. To make sure that the ADRs can be predicted, we filtered out those drugs without features. In total, 766 drugs were filtered and 25% of them were set as a hold-out set. We carried out 5×4 Nested CV in the remaining drug data to evaluate the performance of VKR.

### 2.2 Problem setting

We encoded the drug–ADR pairs data as a binary matrix $Y=[y_{1},y_{2},\ldots ,y_{i},\ldots ,y_{N}]$. Here $y_{i}$ is the ADR vector of drug *i*, with length *M*. *N* is the number of known drugs and *M* is the number of ADRs. In addition, DGI and Chem can also be expressed in the same form, denoted as *X*, where $X=[x_{1},x_{2},\ldots ,x_{i},\ldots ,x_{N}]$ is for known drugs and $x_{i}$ is a feature vector of drug *i* with length *p*. For testing and validation, we randomly selected drugs as the new ones, which were set as validation sets in the CVs and the hold-out set, denoted as $Y^{\text{new}}=[y_{1}^{\text{new}},y_{2}^{\text{new}},\ldots ,y_{i}^{\text{new}},\ldots ,y_{N^{\prime }}^{\text{new}}]$, where $N^{\prime }$ is the number of new drugs. All the ADRs of the selected drugs were masked as 0 simulating the new drugs in the real world, for example $y_{i}^{\text{new}}=0$. The corresponding features were denoted as $X^{\text{new}}=[x_{1}^{\text{new}},x_{2}^{\text{new}},\ldots ,x_{i}^{\text{new}},\ldots ,x_{N}^{\text{new}}]$. We denoted the original drug–ADR matrix data with all drugs included (known drugs and masked new drugs) as $Y_{0}$.

We used DGI as the main feature in this study. This has not been used previously in ADR profile prediction for new drugs. We also studied how all the existing methods work in a commonly used feature—Chem. To compare the ability to integrate features of VKR with others, we also utilized a linear combination of kernels obtained from DGI and Chem as an integrated feature. For the existing methods, features were integrated following the approaches provided by the reviewed paper.

### 2.3 The naïve model

Before we compared with the existing methods, we had designed a naïve method as the baseline. The naïve model uses mean values of ADRs of known drugs to predict the ADRs for all the new drugs. It can be written as the following equation:


(1) $$Y_{ij}^{\text{new}}=\frac{1}{N}\sum_{k}Y_{ik},$$

where $Y_{ij}^{\text{new}}$ is the *i*, *j* entry in matrix $Y^{\text{new}}$, and $Y_{ik}$ is the *i, k* entry of matrix *Y*. As a result, for a given *i*, $Y_{ij}^{\text{new}}$ is a constant for all *j*.

### 2.4 Hybrid of KR and NMF

We developed a method that combines KR and NMF, called VKR. The method performs KR on matrix *V*, where *V* is a matrix factor obtained by an NMF method [Equation (2)], and *V* serves as dependent variables of KR.

**Figure 1.**
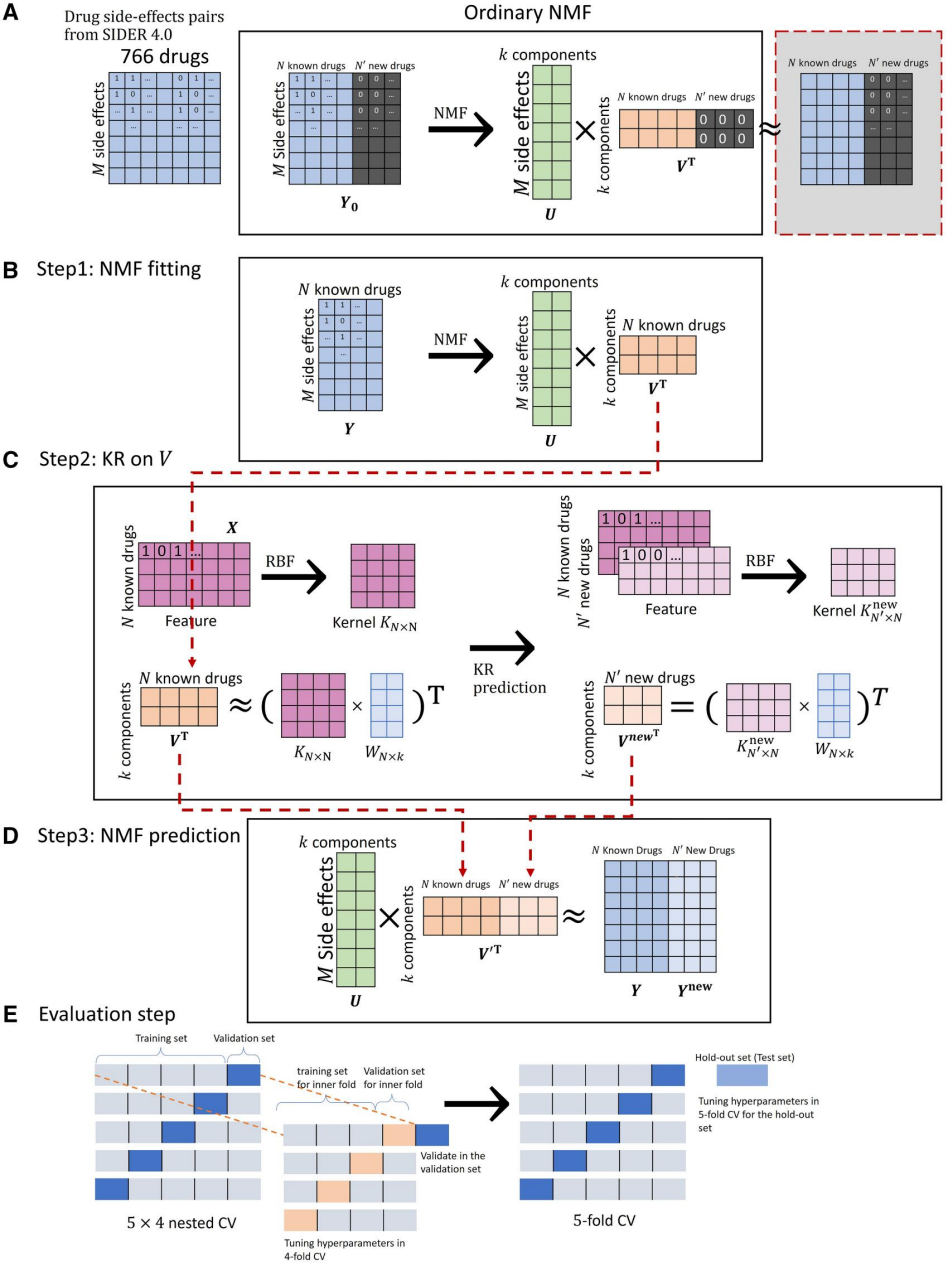
Workflow of VKR. (a) The drug–ADR matrix was derived from SIDER 4.0. Seven hundred and sixty-six drugs were filtered in this study. If a drug has a specific ADR, then the entry was denoted as 1, otherwise, it was denoted as 0. Ordinary NMF cannot be applied for ADR profile prediction because columns for new drugs are set to 0. If $Y_{0}$ is factorized into *U* and $V^{T}$, the columns of $V^{T}$ remain 0, which can only lead to 0 prediction (seen in the dotted line box). (b) In VKR, only the drug–ADR matrix for known drugs was factorized into *U* and $V^{T}$, and $V^{T}$ was used as the dependent variables in KR. (c) Features were transformed into a binary matrix and further transformed into RBF kernel via the approach in Section 2.3. A RBF kernel for known drugs and a RBF kernel for new drugs and known drugs can be obtained according to Section 2.3. The coefficient matrix *W* for $V^{T}$ was trained based on kernel *K*, and a new *V* for the new drugs, $V_{\text{new}}^{T}$ were predicted using the kernel for the new drug and known drugs $K^{\text{new}}$. (d) Then finally back to the prediction step of NMF, $Y^{\text{new}}$ can be predicted by the dot product of the *U* from step 1, and the predicted $V_{\text{new}}^{T}$ from step 2. Here, ${V^{\prime }}^{T}$ is reformed by matrix *V* and $V_{\text{new}}$. And the original $Y_{0}$ can be predicted by the multiplication of *U* and reformed $V^{\prime T}$. (e) Nested CV and CV. VKR was evaluated rigorously by 5×4 Nested CV and hyperparameters were tuned in a 5-fold CV for the hold-out set.

KR was first used in Yamanishi *et al.* (2012) ADR prediction study. It turns out to be Kernel Ridge Regression, which uses a kernel as the independent variable and uses a ridge term to regularize the variable (Supplementary Material 10). NMF is a dimension reduction method, which we applied to de-noise drugs and ADRs into *L* components (Supplementary Table 9). The VKR method consists of two steps. First, we used NMF to obtain two new matrices, by solving the following objective function:


(2) $${\left\|Y-UV^{T}\right\|}^{2},$$

where $Y=[y_{1},y_{2},\ldots ,y_{i},\ldots ,y_{N}]$ is the drug–ADR matrix, $y_{i}$ is the ADR vector of drug *i*, $U=\left[u_{il}\right]\in R^{M\times L}$ is an ADR matrix with *K* new features, $V=\left[v_{jl}\right]\in R^{N\times L}$ is a drug matrix with *L* new features, and U,V are two non-negative matrices (Fig. 1B). Then in the second step, we utilized KR to fit *V* (Fig. 1C), and therefore, the objective function in Supplementary Material 10 (S1) was reformulated into the following equation:


(3) $${\left\|V-KW\right\|}^{2}+\lambda {\left\|W\right\|}^{2},$$

where $K\in R^{N\times N}$ is a kernel matrix, and $W\in R^{N\times K}$ is a coefficient matrix. By minimizing the objective function, *W* can be fitted. λ is a regularization parameter. Gaussian Radial Basis Function (RBF) kernel was used in this study to measure the similarity of two drugs under the same feature. Therefore, entries in K were defined as an inner product $k_{i,j}=k(x_{i},x_{j})=\text{exp}({\left\|x_{i}-x_{j}\right\|}^{2}/2\sigma^{2})$, where σ is a width parameter, and $x_{i}\in R^{p}$ is a feature vector of drug *i*.

And the prediction of *V* can be calculated by the following equation:


(4) $$V^{\text{new}T}=K^{\text{new}}V^{T},$$

where $V^{\text{new}}=\left[v_{jk}^{\text{new}}\right]\in R^{N^{\prime }\times K}$ represents the new *k* features of new drug *j*, and $K^{\text{new}}\in R^{N^{\prime }\times N}$ was defined as $k_{i,j}^{\text{new}}=k(x_{i}^{\text{new}},x_{j})=\text{exp}({\left\|x_{i}^{\text{new}}-x_{j}\right\|}^{2}/2\sigma^{2})$, where $x_{i}^{\text{new}}\in R^{p}$ is a feature vector of new drug *i*.

In the last step of VKR, we calculated the approximation of matrix $Y_{\text{new}}$ by matrix multiplication. Together with *U*, we factorized from NMF, we constructed a prediction matrix for new drugs, $Y_{\text{new}}=UV^{\text{new}T}=U{\left(K^{\text{new}}W\right)}^{T}$ (Fig. 1D).

### 2.5 Evaluation

To evaluate the performance of VKR, we held out 25% of the drugs as a hold-out set, and the remaining data were used in 5×4 Nested CV. AUPR and AUROC were used as the performance metrics in our study. We then ran an ordinary CV to tune the hyperparameters for the hold-out set. The hyperparameters to be tuned for VKR are *k* (the number of components of NMF, tuned with the set of hyperparameters {5,10,15,20,25}), λ (the regularization level of ridge term of KR, tuned with the set of hyperparameters $\left\{\ldots ,10^{-2},10^{-1},10^{0},10^{1},10^{2},\ldots \right\}$), and σ (the width of the kernel, tuned with the set of hyperparameters $\left\{\ldots ,10^{-2},10^{-1},10^{0},10^{1},10^{2},\ldots \right\}$). In the ordinary 5-fold CV, the drugs in the training set were split into five groups. In a single fold, one of the groups was set as a validation set, and the rest of them were combined as a training set. This process was repeated five times until all groups were used as a validation set once (Fig. 1E, 5-fold CV). In 5×4 Nested CV, an additional 4-fold CV was run in the training set of each fold (Fig. 1E, 5×4 Nested CV). Different combinations of hyperparameters were tested within these four inner folds (folds in 4-fold CV). The best combinations were chosen as the hyperparameters for the validation set of each fold. In this way, models were not underestimated or overestimated. To make the models comparable, we applied the same CV strategies to all the other methods (Fig. 1E).

### 2.6 Review and comparison

We compared VKR and the naïve method with a representative collection of existing methods, for the reason that comparison of all existing methods is time-consuming. Some of the classic methods were outperformed by the latest proposed one, others might not have significant improvement compared with the representative methods; furthermore, methods with additional strategies affect the results of comparison. For example, Lee *et al.* (2017) applied NB, KNN, and RF separately to subsets of drug–ADR data. Performance was greater than SVM. However, using NB, KNN, and RF separately without this strategy was outperformed by SVM (Liu *et al.* 2012)—SVM might have similar improvement by applying additional strategies.

Therefore, we selected the comparator methods using defined rules (Supplementary Material 12) in order to cover most of the existing methods. First, we considered the advanced methods proposed that outperformed the others in recent years; then we removed additional strategies on data (Supplementary Material 12); thirdly, if the methods have equivalent performance, we only considered one of them (Supplementary Material 12). Following these rules, we found that parts of the 20 advanced methods are worth studying (Table 2, a collection of existing studies on the ADR profile prediction problem to the best of our knowledge is in Supplementary Table 6).

We set a random seed for the 5×4 Nested CV. As a consequence, for each fold, methods were run in the same subset of the training data and were validated with the same validation set. This allowed us to use paired *t*-tests to evaluate the statistical significance of the differences in means of AUPRs and AUROCs across the five folds of the CV (Supplementary Tables 2 and 3).

## 3 Results

### 3.1 Most existing methods fail to outperform a naïve method

We evaluated a wide range of methods for the problem of side effect profile prediction, using data from SIDER 4.1 data. As a baseline against which to compare method performance, we devised a naïve model that assigned side effects to drugs according to the proportion of all drugs with that side effect (see Section 2 for details). Surprisingly, we found that the naïve model achieved good performance, with AUPR and AUROC reaching 0.37 and 0.91, respectively, in 5×4 Nested CV (Table 1). Indeed, most of the sophisticated methods we evaluated failed to achieve any improvement in AUPR over the naïve model, and the naïve model was the top-performing method as evaluated by AUROC. The naïve model does not use any features for prediction. For the other methods, we utilized DGI and Chem as the drug features to make predictions for ADRs. Here we provide the results using DGI as a main example (Table 1).

**Table 1.** The main results of the existing methods using DGI as a feature

	5×4 Nested CV				Hold-out set	
	AUPR	*P*-value	AUROC	*P*-value	AUPR	AUROC
Naïve	0.37 ± 0.01		**0.91** ± 0.00		0.36	**0.91**
KR	**0.43** ± 0.01	4.4e-4	0.89 ± 0.00	3.8e-4	**0.41**	0.89
LNSM-RLN	0.32 ± 0.01	4.9e-4	0.87 ± 0.01	1.9e-3	0.25	0.88
LNSM-Jaccard	0.29 ± 0.01	1.3e-4	0.89 ± 0.00	5.1e-4	0.35	0.91
SVM-RBF	0.38 ± 0.01	1.2e-1	0.84 ± 0.01	9.7e-5	0.38	0.85
SVM-WGTS	0.24 ± 0.01	7.3e-6	0.69 ± 0.01	1.3e-6	0.24	0.68
VKR	**0.43** ± 0.01	4.4e-4	**0.92** ± 0.00	4.0e-4	**0.41**	**0.91**

The results of VKR were listed to compare with the existing methods. *P*-values are for the paired *t*-test compared with the naïve model. Top values of Nested CV are bolded. The corresponding values in the hold-out set were also bolded, meaning that two validations show similar results. The bolded values in row VKR shows our method reached the top performance.

**Table 2.** Review of existing studies on ADR profile prediction and their model comparison

Study	Methods used	Methods comparison
Pérez-Nueno *et al.*(2015)	ET	ET≈(RF, SVM, LR, KNN)>NB
Hammann *et al.*(2010)	OCCA, SCCA	SCCA≈**SVM-RBF**> OCCA>KNN
Zhang *et al.*(2017)	LNSM, LNSM-SMI	(**LNSM, LNSM-SMI**)>OCCA, SCCA
Yamanishi *et al.*(2012)	KR, MKR	(**KR, MKR**)>OCCA
Jiang *et al.*(2020)	SVM-WGTS, SVM-GTS	**SVM-WGTS** ≈ SVM-GTS>LNSM> (SVM-RBF, SVM-polynomial, SVM-linear, RR)
Zhang *et al.*(2016)	LNSM, LNSM-SMI, LNSM-CMI	(**LNSM, LNSM-SMI, LNSM-CMI**)>(OCCA, SCCA, SVM-RBF, FS-MLKNN)
Seo *et al.*(2020)	RF, stacking of (RF, LR, NB, XGBoost)	RF≈stacking of (RF, LR, NB, XGBoost)
Liu *et al.*(2012)	SVM-RBF	**SVM-RBF** > (LR, NB, KNN, RF)
Lee *et al.*(2017)	(NB, KNN, RF) in three ADRs frequency intervals, NB, KNN, RF	(NB, KNN, RF) in three ADRs frequency intervals>SVM-RBF>RF>(OCCA, SCCA)
Zhou *et al.*(2020)	Boosted RF (BRF, MEDICASCY)	BRF≈**LNSM-SMI**> FSMLKNN> multi-layer perceptron>KNN> KG-SIM-PROP>K-means>RF

‘Methods used’ are the methods proposed in the relative studies. ‘Methods comparison’ are the methods compared in their study. ‘≈’ means the methods have close performance, and ‘>’ stands for the methods on the left outperform the right ones; the bold text are the methods we reviewed. This table only gives a brief idea of the relationship between methods. The truth might be more complex since methods were evaluated by multiple metrics. A more comprehensive summary is in Supplementary Table 6.

**Figure 2.**
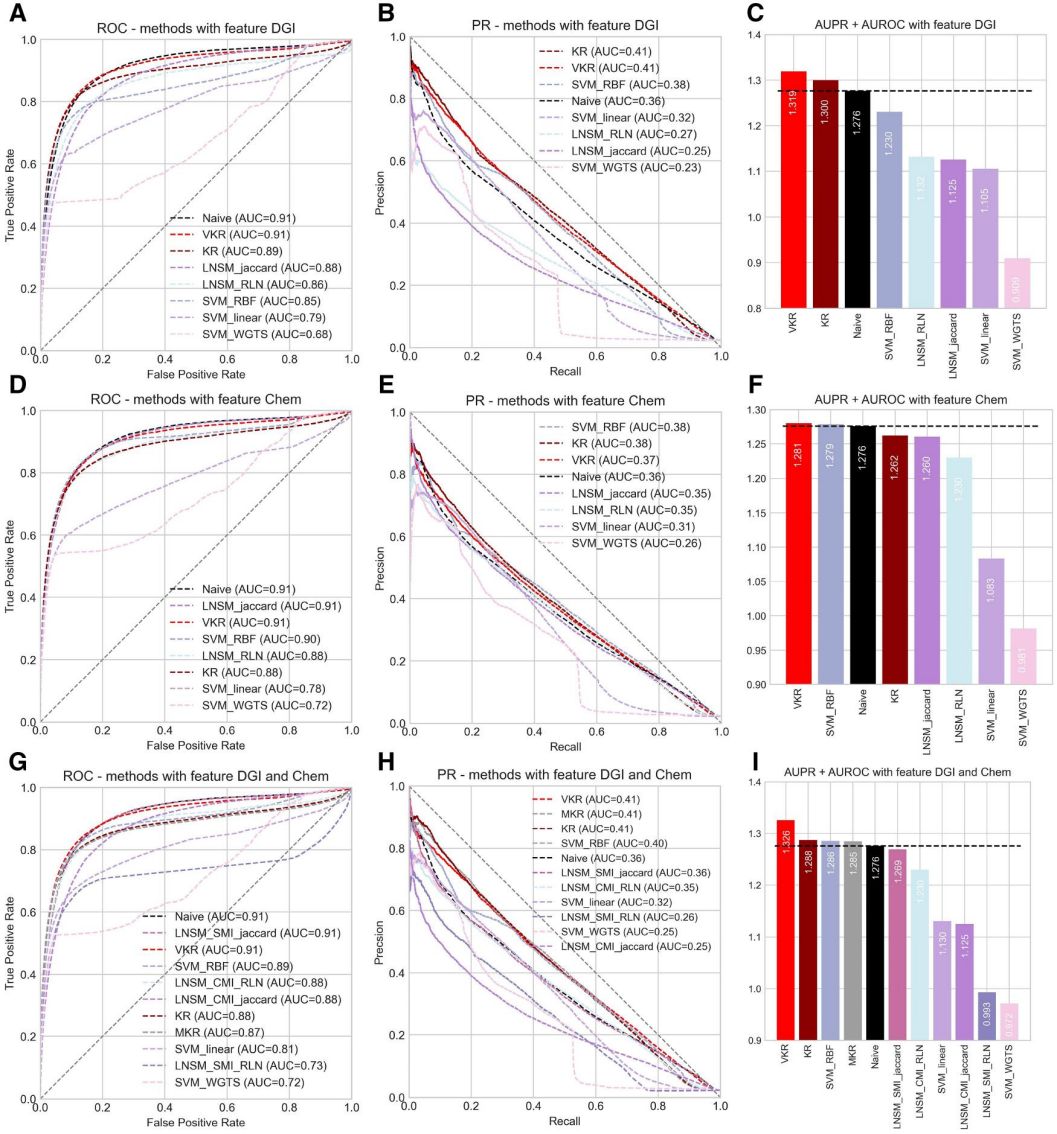
Results of the hold-out set for all methods. (a) AUROC of all the methods with the feature DGI. The naïve method and VKR had the same level of AUROC, outperforming the other methods. (b) KR and VKR reached a similar level of AUPR (Nested CV, *P* = .57), 0.41 and 0.41, respectively, higher than the existing methods. (c) We also added AUPR and AUROC together. This metric provides a summary of the overall performance of the methods. VKR ranked first, showing that VKR reached good performance in both metrics. (d) AUROC of all methods with the feature Chem. naïve and VKR ranked at the top, which was the same as the result in Nested CV (*P* = .54). (e) AUPR of all methods with feature Chem. VKR reached an AUPR of 0.37, exceeded by KR and SVM-RBF. (f) VKR ranked the top in the overall metric. (g) The top AUROC with integrated features was obtained by naïve (0.91), VKR (0.91), and LNSM-SMI with Jaccard similarity (0.91). (h) VKR, KR, and MKR achieved the top AUPR. (i) For the integrated features, VKR had an overall score greater than the others.

The KR method had the highest AUPR using the DGI features (AUPR=0.43), with a large gap in this metric to the second best method, SVM; however, the AUROC was higher for the naïve method (Table 1). The results reflect that KR might have a strong ability to find true-positive cases in the minority data but have a shortcoming that cannot distinct positive and negative as well as the other methods. However, AUPR may be more relevant than AUROC when the data are highly imbalanced, and at the same time, it may reveal the poor performance of some methods whose AUROC is high (Saito and Rehmsmeier 2015). This suggested that KR might be a better method than others with a slightly higher AUROC and that a modification of KR may have the potential to provide an improvement in AUROC while retaining the high AUPR. KR with feature Chem or DGI+Chem as input shared the same tendency (Fig. 2D, E, G, and H; Supplementary Table 1).

### 3.2 Evaluation of VKR

We propose a modification of KR that first applies NMF to de-noise the input data and then applies KR on the drug-related non-negative matrix factor resulting from the factorization (see Section 2 for a detailed description). After running 5×4 Nested CV, VKR reached a result of 0.43 in AUPR applying DGI as a feature, which was the same as KR (*P* = .57; the AUPR and AUROC for each fold of Nested CV can be seen in Supplementary Tables 7 and 8). At the same time, this model obtained the highest AUROC, 0.92 (higher than KR, *P* < .05) with DGI (Table 3). Thus, VKR worked as well as KR in predicting positive cases—the minority cases, in ADR profile prediction, while also achieving the highest AUROC. This suggests that VKR can also predict well in TN cases, resolving a potential disadvantage of KR.

**Table 3.** Comparison between KR and VKR under DGI, Chen, and DGI + Chem

	5×4 Nested CV				Hold-out set	
	AUPR	*P*-value	AUROC	*P*-value	AUPR	AUROC
${\text{KR}}_{\text{DGI}}$	0.43 ± 0.01	5.7e-1	0.89 ± 0.00	2.7e-5	0.41	0.89
${\text{VKR}}_{\text{DGI}}$	0.43 ± 0.01		**0.92** ± 0.00		0.41	**0.91**
${\text{KR}}_{\text{Chem}}$	0.40 ± 0.01	1.6e-1	0.89 ± 0.01	1.5e-2	0.38	0.88
${\text{VKR}}_{\text{Chem}}$	0.40 ± 0.01		**0.91** ± 0.01		0.37	**0.91**
${\text{KR}}_{\text{DGI}+\text{Chem}}$	0.43 ± 0.01	4.3e-1	0.90 ± 0.01	6.6e-2	0.41	0.88
MKR	0.44 ± 0.01	1.6e-1	0.89 ± 0.01	2.9e-3	0.41	0.87
${\text{VKR}}_{\text{DGI}+\text{Chem}}$	0.44 ± 0.02		**0.91** ± 0.01		0.41	**0.91**

MKR is an extended version of KR for integrating features only, so MKR was only used in feature DGI + Chem. *P*-values are for the paired *t*-test compared with the VKR. Bold text shows VKR outperforms KR in AUROC.

The use of DGI as a feature increased the performance of VKR, compared with using Chem as a feature (Table 3;Supplementary Fig. 4). A slightly weaker performance was recorded under feature Chem (AUPR=0.40), but it still outperformed the existing methods under the same feature (Supplementary Table 1 and Fig. 5, and *P*-value in Supplementary Tables 2 and 3). DGI, which contains information on genes, might be a better feature for predicting ADRs than Chem. In addition, we studied the ability to integrate features with VKR, using a linear combination of two kernels, which were obtained from feature Chem and DGI. This gave a slight improvement compared to DGI alone but did a better job than feature Chem. In other words, the integration of features increases the ability to predict ADRs, and DGI contributed more to the combination of features in VKR.

### 3.3 Evaluation on the hold-out set

Lastly, the methods were evaluated on the hold-out set. From the results of 5×4 Nested CV, we had already observed that VKR can obtain the same AUPR level and exceeded the AUROC of KR. In the hold-out set, the AUPR of VKR and KR were almost identical, while the AUROC of VKR was far better than KR, in agreement with the results obtained using Nested CV. For the hold-out set, we also studied the Area Under Curves (AUCs) for all the reviewed methods with feature DGI, Chem as well as the integration of them (Fig. 2). We used the sum of AUPR and AUROC as an overall summary of performance and, again, VKR showed the best performance of all the methods evaluated. VKR with integrated features reached the highest AUPR (0.41) in the test set. VKR based on DGI and DGI+Chem had better performance than that based on Chem. Furthermore, there was a greater gap in AUPR between KR and VKR compared to the next best methods when using DGI for prediction (Fig. 2A–C) than when Chem was used (Fig. 2D–F).

Although some methods had slightly greater AUPR or AUROC (Fig. 2), no method offered a statistically significant improvement in either metric over VKR. For example, the AUPR of VKR under feature Chem (0.37) was just under the AUPR of KR (0.38; Fig. 2D); however, the difference was not significant across the folds of the Nested CV (*P*-values = .16 from a paired *t*-test), and VKR provided significant improvement (*P* < .05) over KR on the other metric. Similarly, VKR, the naïve method, and LNSM-Jaccard were the top-performing methods for AUROC under feature Chem, while VKR showed significantly higher AUPR than the other two methods (Fig. 2D and E; SVM-RBF reached a significantly lower AUPR in Nested CV, though it gave the best performance on the test data set). Therefore, in each case, VKR provided a significant improvement (*P* < .05) over the comparator method on the other metric.

## 4 Discussion

### 4.1 Imbalanced data and metric selection

In this study, a method that attains a good result will be high in both AUPR and AUROC. We found that the ADR data are highly imbalanced. In the drug–ADR matrix, only 2.44% of entries are assigned as one. In other words, the number of negative cases is far greater than the number of positive cases. However, the positive cases hold greater significance than the negative cases for application purposes. In the context of drug development, it may be more important to be able to predict the ADRs that may occur, rather than the ADRs that are not likely to occur. This means that we should pay more attention to precision (TP/(TP+FP)) and recall (TP/(TP+FN)) curves rather than the TN cases. Therefore, AUPR is a metric that does not consider TN. With AUPR, the ability to identify true positives in the minority (positive) cases is not overestimated since the imbalanced data biased to negatives might lead to high TN. AUPR contains more relevant information than AUROC in imbalanced datasets, revealing the poor performance of some methods despite a high AUROC (Jeni *et al.* 2013; Saito and Rehmsmeier 2015). Therefore, AUPR is more valuable as an evaluation metric and we considered it as the criterion to be optimized in Nested CV and the ordinary CV. Nevertheless, AUROC reveals the ability of models to separate positive cases and negative cases (false-positive rate (FP/(FP+TN)) to true-positive rate (TP/(TP+FN))). Thus, we considered AUPR and AUROC simultaneously, and we summed these two metrics up directly as a new metric (AUROC+AUPR) to give an overall evaluation.

### 4.2 A more reliable and unbiased estimation of performance

Here, 5×4 Nested CV was used to obtain an unbiased estimate of performance of the classification scheme, whereas most existing studies have used K-fold CV or Leave-one-out CV (LOOCV). Of all the existing studies that we have reviewed, 15 used K-fold CV; 3 of them used LOOCV; and none of them used a nested CV and a hold-out set to validate their results (Supplementary Table 6). The results of the existing method might not be reliable, because hyperparameters were tuned in the K-fold CV or LOOCV, which means they ran the validation sets in K-fold CV or LOOCV multiple times for tuning (Cawley and Talbot 2010). Then information might leak into the model and result in overfitting. Nested CV can avoid the shortcoming caused by K-fold CV or LOOCV (Cawley and Talbot 2010). In addition to the use of Nested CV, we applied paired *t*-tests over a fixed set of data partitions during CV as well as a hold-out test set to guarantee reliable and unbiased estimation of performance.

### 4.3 Problem of existing methods

The majority of the current methods exhibit a lack of consistent superiority in AUPR and AUROC over the naïve method. This means the existing methods lack the ability to find a balance between high in the AUROC—achieving effective separation of positive and negative cases, and high in the AUPR—priorly and effectively identifying the true positive within the positive cases.

In Zhang *et al.* (2016), the family of LNSM performed better than SVM, LR, NB, KNN, and RF (Liu *et al.* 2012), SCCA (Pauwels *et al.* 2011) and OCCA (Mizutani *et al.* 2012). We chose a more advanced similarity, regularized linear neighbour (RLN) similarity (Zhang *et al.* 2016) to calculate the nearest neighbours in the review of LNSM. First, the size of the data might affect the performance of LNSM (Zhang *et al.* 2016). Six hundred nearest neighbours of RLN similarity (600-RLN) of LNSM (LNSM-RLN) provided the best performance, and the lower number of nearest neighbours reduced the performance of LNSM (Zhang *et al.* 2016). However, 200-RLN was used in our study since 200 drug neighbours almost reached the largest number of observations in the inner fold of 5×4 Nested CV. So for the purpose of comparison, we also reviewed Jaccard similarity in LNSM (LNSM-Jaccard), which is the best among the similarities that are not affected by the size of the sample. Secondly, overfitting might hide the true performance of the earlier study. The results of LNSM in K-fold CV might be overestimated in Zhang *et al.* (2016) since there is a gap between the results from K-fold CV and the hold-out set, while both results of LNSM from Nested CV and hold-out set in our study are close to the hold-out set provided in Zhang *et al.* (2016). Based on the unbiased result in the review, the family of LNSM might not offer an improvement over the naïve approach to ADR profile prediction (*P*-value in Supplementary Tables 2 and 3).

SVM has been particularly widely used for ADR profile prediction. The performance of SVM may depend on data pre-processing steps (Huang *et al.* 2013, Shaked 2016, Lee *et al.* 2017). However, for a fair comparison, we did not apply any pre-processing steps to the data, since we might achieve similar improvements in other models after pre-processing. The RBF kernel was used in our study, which was also commonly used in other studies (Pauwels *et al.* 2011, Liu *et al.* 2012). SVM-RBF was found to have an AUPR of 0.38, which was slightly higher than the naïve method and an AUROC of 0.87, which was significantly lower than the naïve method (Table 1). However, SVM cannot solve the problem that observations belong to multiple categories, so it requires fitting the coefficients multiple times for each ADR. Because there were 5599 ADRs in total, SVM was far more time-consuming than the naïve method. RBF was found not to be the best kernel for SVM, and a weighted generalized T-Student kernel (WGTS) kernel has been developed (Jiang *et al.* 2020). However, we discovered that SVM-WGTS, based on DGI, performed more poorly than the SVM-RBF and even worse than the naïve method after 5×4 Nested CV (Table 1).

### 4.4 KR is the potential to be improved

Of all the existing methods, KR showed a good ability to identify the true positives within the positive class (AUPR). However, it had a relatively weaker capability to effectively discriminate between positive and negative cases (AUROC); and surprisingly, the naïve method was best in this respect, suggesting that existing methods are not satisfactory. The naïve method also required the least computational resources and time. Some methods are time-consuming (e.g. SVM). Others might exhibit a slightly improved AUROC compared with KR but their performance still falls short of the naïve method. Thus, KR, with the highest AUPR, was considered worth further investigation for the study of ADR profile prediction.

### 4.5 Necessity of combining KR and NMF

The nonlinearity and the kernel trick introduced in KR helped it find patterns in the minority of imbalanced data. KR can be seen as a regression with kernel trick on its explanatory variables. The kernel trick is to project the variables to a higher dimension without knowing the exact function, but only by calculating the inner product between data points. It enables us to fit the unknown nonlinear pattern of drug–ADR data in high-dimensional, implicit feature space. This property allows KR and VKR to find patterns for the minority (positive) cases from higher dimensions, leading to greater AUPR scores than linear methods, including the naïve method. Furthermore, the regularization term in KR allows us to reduce the variance of prediction. However, besides the sparsity and the imbalance, the substantial amounts of noise in the ADR data cause high prediction bias in the ADR profile prediction. KR using existing ADR data as dependent variables may suffer from the high noise level in the data. We thus considered to de-noise the ADR data first and then used the de-noised data as the new dependent variable for KR.

NMF is a well-studied dimension reduction method (Supplementary Material 10). Similar to the naïve method we designed, NMF does not require extra feature data to make predictions or de-noise when some ADRs (entries of the drug–ADR matrix in Fig. 1A) are missing. However, in the study of ADR profile prediction, it cannot make predictions for new drugs (missing columns of the drug–ADR matrix in Fig. 1A) but can help to reduce noise via factorization and reconstruction of the matrix. This property of noise reduction further improves the ability to distinguish between the positive cases and the negative cases of KR and results in a higher AUROC of VKR. In short, NMF is a potential way to improve KR for ADR profile prediction and, potentially, also for other applications.

### 4.6 Feature DGI, Chem, and the integration of them

In the process of reviewing the existing methods, we found that DGI was never used in ADR profile prediction for new drugs (but had been used in finding hidden ADRs for known drugs). DGIdb was mined from multiple databases including DrugBank, PharmGKB, Chembl, and others. Therefore, we assumed that DGI is a potential gene-related feature that contains comprehensive information to restore the mechanism of ADRs, and might further improve the performance of ADR profile prediction. We also compared the existing methods under a commonly used feature, Chem. As a result of the review, adopting feature Chem showed a similar top ranking to predictions with feature DGI—KR obtained the highest AUPR but was exceeded by others in AUROC (Supplementary Table 1 and *P*-value in Supplementary Tables 2 and 3). Although KR achieved the highest AUPR when applied to Chem, the difference in AUPR performance compared to other methods was not substantial. In contrast, when KR was paired with DGI, there was a notable disparity in AUPR performance, indicating a significant performance gap compared to the other methods. In Section 3, VKR also showed a similar tendency, supporting the assumption that DGI is a potential feature for ADR profile prediction.

In order to mitigate the complexity of VKR, we opted for the linear combination of the kernels in KR instead of employing MKR. Yamanishi *et al.* (2012) developed two approaches to integrate features. One simply uses a linear combination of the kernels as the integration method to combine two features (Supplementary Material 10 and Supplementary Table 4); the other, called MKR, utilizes a linear combination of two prediction scores as a new prediction score for integrated features (Supplementary Material 10 and Supplementary Table 5). MKR is controlled by two ridge terms, while KR has one ridge term only—MKR introduces another hyperparameter (Supplementary Material 10 and Supplementary Tables 4 and 5). Our review shows that both of Yamanishi’s integrated features-based methods showed improvement compared with the single-feature version (DGI or Chem; Supplementary Tables 1 and 5 and *P*-value in Supplementary Tables 2 and 3). MKR shows a better performance compared with KR with integrated features (DGI+Chem). For VKR, it is possible that using MKR on *V* might achieve a better result. However, as we improved KR with NMF, we had already introduced a new hyperparameter *k*. Adopting MKR in the VKR will introduce another hyperparameter and further increase the cost of training. Therefore, for VKR with integrated features, we used a linear combination of kernels in the KR step. Nonetheless, this integration approach in VKR reached the highest AUPR over all the methods and features in our study.

## 5 Conclusion

In this study, we designed the naïve method as a baseline for the first time for method comparison of ADR profile prediction. For a more reliable and unbiased estimation of performance, we performed a comparison of ADR prediction methods using a rigorous evaluation procedure. The 5×4 Nested CV was used for the first time to evaluate the ADR profile prediction method; in addition, a hold-out set was separated from the dataset to validate the conclusion from Nested CV, providing an unbiased evaluation. AUPR was considered the main metric because it is well-suited to imbalanced data with a very small proportion of positive cases. AUROC was used as the supporting metric as it reflects the ability to divide positive and negative cases.

Surprisingly, our study suggests that the naïve method is a competitive method with a small amount of time and computational consumption, questioning the value of existing advanced methods. None of the existing methods could consistently surpass the performance of the naïve method in all respects. Among all these methods, KR had the best performance among all the existing methods, but it did not effectively separate positive and negative cases, as evaluated by AUROC. Therefore, we decided to enhance KR further in order to optimize its performance. We improved KR to a hybrid method that combines NMF and KR, VKR. It ranks the best in the overall metrics (AUPR, AUROC, and AUROC+AUPR).

In conclusion, VKR can balance the ADR profile prediction of distinguishing the positive and negative cases against the ability to effectively detect the rare true-positive cases. Hence, VKR solves the disadvantage of KR, that KR functions well in predicting minority cases, sacrificing the performance in negative cases. And VKR shows the same advantage when adopting DGI, Chem, and integrated features. Our results also suggest that DGI might be a better feature for ADR prediction.

In the future, we will investigate the specific ADRs of drugs in the hold-out set to further support the prediction made by VKR. In addition, more features will be introduced to increase the performance. Finally, with some pre-processing steps, such as feature selection, we might be able to make further progress in VKR.

## Supplementary Material

vbae009_Supplementary_Data

## Data Availability

The code for VKR and the reviewed methods are available as Jupyter Notebook. Results of figures, tables, and supplementary can be reproduced by data and code provided at https://github.com/YezhaoZhong/VKR, with all required Python modules listed. SIDER can also be downloaded from http://sideeffects.embl.de/. DGIdb is also available at https://www.dgidb.org/. The Chemical structure fingerprints are available at https://pubchem.ncbi.nlm.nih.gov/.
