# Supplementary material for: Non-Negative matrix factorization combined with kernel regression for the prediction of adverse drug reaction profiles
Source: Bioinform Adv. 2024 Jan 23;4(1):vbae009. doi: 10.1093/bioadv/vbae009 (PMC11087822; doi:10.1093/bioadv/vbae009)
Supplement: vbae009_Supplementary_Data [file vbae009_supplementary_data.pdf]

Supplementary 1

We reviewed all the existing methods for the ADRs prediction with feature DGI and Chem as input. The AUROC of the Naïve method is outstanding. It is observed that KR and VKR reach the top AUPR in the nested CV, and VKR also reaches the top in the AUROC overall method we compared. A similar pattern is also observed in the hold-out set. For feature Chem, KR and VKR also is ranked the top in AUPR while VKR is also ranked the top in AUROC. For the hold-out set, SVM-RBF slightly outperforms VKR, leading us to consider that the SVM-RBF might have the same level of performance in AUPR. And like DGI, the integrated feature of DGI and Chem shows that VKR has the same level of AUPR as the KR family, but a higher AUROC is shown.

Table S 1 Model Comparison

	5*4 nested CV				hold-out set	
	AUPR	std	AUROC	std	AUPR	AUROC
Naïve	0.3715	0.0059	**0.9082**	0.0016	0.3635	0.9127
	Feature DGI					
KR	**0.4348**	0.0097	0.8924	0.0022	0.4112	0.8888
LNSM-RNL	0.3182	0.0065	0.8695	0.0116	0.2467	0.8787
LNSM-jaccard	0.2891	0.0100	0.8893	0.0032	0.3508	0.9095
SVM-WGTS	0.2438	0.0076	0.6859	0.0098	0.2374	0.6784
SVM-RBF	0.3802	0.0074	0.8355	0.0091	0.3778	0.8526
SVM-linear	0.3353	0.0130	0.7957	0.0115	0.3154	0.7899
VKR	**0.4336**	0.0081	**0.9160**	0.0023	0.4080	0.9112
	Feature Chem					
KR	**0.3957**	0.0104	0.8898	0.0134	0.3806	0.8813
LNSM-RNL	0.3462	0.0111	0.8652	0.0088	0.3486	0.8815
LNSM-Jaccard	0.3663	0.0037	**0.9078**	0.0022	0.2467	0.8787
SVM-linear	0.3149	0.0117	0.7811	0.0149	0.3078	0.7756
SVM-WGTS	0.2726	0.0106	0.7594	0.0180	0.2367	0.6676
SVM-RBF	0.3870	0.0067	0.9012	0.0055	0.3807	0.8981
VKR	**0.3981**	0.0132	**0.9098**	0.0062	0.3739	0.9066
	Feature DGI and Chem					
MKR	**0.4421**	0.0090	0.8905	0.0094	0.4138	0.8713
KR	**0.4315**	0.0111	0.8954	0.0146	0.4125	0.8753
LNSM-CMI-RNL	0.3462	0.0111	0.8652	0.0088	0.3486	0.8815
LNSM-SMI-RNL	0.2949	0.0184	0.7378	0.0132	0.3572	0.9122
LNSM-CMI-Jaccard	0.2891	0.0100	0.8893	0.0032	0.2467	0.8787
LNSM-SMI-Jaccard	0.3729	0.0031	**0.9107**	0.0024	0.3572	0.9121
SVM-WGTS	0.2513	0.0120	0.7515	0.0230	0.2542	0.7179
SVM-RBF	0.4039	0.0130	0.8997	0.0063	0.3963	0.8892
SVM-linear	0.3406	0.0162	0.8048	0.0160	0.3249	0.8055
VKR	**0.4389**	0.0187	**0.9105**	0.0082	0.4147	0.9116

Supplementary 2

Table S 2 P-value of AUPR

	Naive	KR-DGI	KR-Chem	MKR	KR-DGI+Chem	LNSM-RLN-DGI	LNSM-RLN-Chem	LNSM-CMI-RLN	LNSM-SMI-RLN	LNSM-Jaccard-DGI	LNSM-Jaccard-Chem	LNSM-CMI-Jaccard	LNSM-SMI-Jaccard	SVM-WGTS-DGI	SVM-WGTS-Chem	SVM-WGTS-DGI-Chem	SVM-RBF-DGI	SVM-RBF-Chem	SVM-RBF-DGI+Chem	SVM-linear-DGI	SVM-linear-Chem	SVM-linear-DGI-Chem	VKR-DGI	VKR-Chem	VKR-DGI+Chem
Naive	-	0	0.005	0	0.001	0	0.001	0.001	0.002	0	0.171	0	0.638	0	0	0	0.118	0.006	0.006	0.012	0.001	0.023	0	0.013	0.044
KR-DGI	-10.674	-	0.004	0.001	0.593	0	0	0	0	0	0	0	0	0	0	0	0	0.001	0.003	0	0	0	0.568	0.008	0.309
KR-Chem	-5.721	5.828	-	0.002	0.001	0	0	0	0	0	0.009	0	0.018	0	0	0	0.03	0.014	0.107	0.002	0	0.001	0.004	0.162	0.173
MKR	-12.073	-8.738	-7.122	-	0.108	0	0	0	0	0	0	0	0	0	0	0	0	0	0.001	0	0	0	0.007	0.003	0.159
KR-DGI+Chem	-9.639	0.58	-8.995	2.062	-	0	0	0	0	0	0.001	0	0.001	0	0	0	0	0	0.002	0	0	0	0.701	0	0.428
LNSM-RLN-DGI	10.376	16.061	11.283	18.579	17.968	-	0.017	0.017	0.05	0.004	0	0.004	0	0	0.003	0.001	0	0	0.001	0.065	0.659	0.079	0	0.001	0.005
LNSM-RLN-Chem	8.582	11.234	14.805	12.271	13.108	-3.956	-	nan	0.009	0.002	0.023	0.002	0.008	0	0	0	0.004	0	0.001	0.339	0.005	0.566	0	0.001	0.012
LNSM-CMI-RLN	8.582	11.234	14.805	12.271	13.108	-3.956	nan	-	0.009	0.002	0.023	0.002	0.008	0	0	0	0.004	0	0.001	0.339	0.005	0.566	0	0.001	0.012
LNSM-SMI-RLN	7.524	15.947	10.568	18.255	22.443	2.779	4.738	4.738	-	0.558	0.002	0.558	0.001	0.016	0.115	0.018	0	0.001	0	0.001	0.039	0.003	0	0	0.001
LNSM-Jaccard-DGI	14.658	20.539	11.841	22.575	16.502	6.116	7.157	7.157	0.638	-	0	nan	0	0.005	0.139	0.023	0	0	0	0.003	0.043	0.009	0	0	0.002
LNSM-Jaccard-Chem	1.667	11.128	4.761	13.067	9.856	-19.034	-3.573	-3.573	-7.616	-20.83	-	0	0	0	0	0	0.037	0.011	0.008	0.013	0.002	0.052	0	0.012	0.04
LNSM-CMI-Jaccard	14.658	20.539	11.841	22.575	16.502	6.116	7.157	7.157	0.638	nan	20.83	-	0	0.005	0.139	0.023	0	0	0	0.003	0.043	0.009	0	0	0.002
LNSM-SMI-Jaccard	-0.508	10.426	3.879	12.383	9.198	-20.912	-4.918	-4.918	-8.351	12.383	-18.694	-21.125	-	0	0	0	0.168	0.031	0.014	0.006	0.001	0.024	0	0.021	0.056
SVM-WGTS-DGI	30.063	30.532	25.036	31.089	23.507	11.334	16.436	16.436	4.007	5.717	24.96	5.717	27.76	-	0.007	0.253	0	0	0	0.001	0.001	0.001	0	0	0.001
SVM-WGTS-Chem	27.204	20.243	53.146	21.509	26.575	6.651	33.951	33.951	2.006	1.846	16.027	1.846	17.961	-5.201	-	0.003	0	0	0	0.003	0.001	0.001	0	0	0.001
SVM-WGTS-DGI-Chem	21.437	24.244	59.309	25.613	34.129	8.515	18.863	18.863	3.864	3.568	15.792	3.568	17.498	-1.333	6.69	-	0	0	0	0.001	0	0	0	0	0
SVM-RBF-DGI	-1.983	14.791	3.299	19.766	15.939	-12.279	-5.881	-5.881	-13.495	-15.755	-3.079	-15.755	-1.681	-20.345	-17.762	-20.072	-	0.131	0.004	0	0	0.002	0	0.008	0.043
SVM-RBF-Chem	-5.447	9.452	4.174	11.218	10.666	-11.925	-11.39	-11.39	-9.762	-13.043	-4.461	-13.043	-3.269	-32.622	-38.077	-41.448	-1.892	-	0.014	0.002	0	0.002	0.001	0.033	0.09
SVM-RBF-DGI+Chem	-5.356	6.331	-2.075	7.943	7.715	-10.105	-9.677	-9.677	-13.421	-12.261	-4.886	-12.261	-4.179	-20.433	-21.852	-28.352	-6.041	-4.207	-	0	0	0	0.005	0.743	0.215
SVM-linear-DGI	4.346	20.802	7.234	26.441	18.87	-2.527	1.084	1.084	-8.324	-6.198	4.257	-6.198	5.241	-9.594	-6.297	-8.816	10.661	7.027	10.438	-	0.029	0.391	0	0	0.003
SVM-linear-Chem	9.641	19.23	25.424	22.013	85.844	0.475	5.574	5.574	-3.031	-2.927	7.566	-2.927	8.867	-8.796	-8.094	-13.014	18.549	18.624	28.852	3.323	-	0.004	0	0	0.001
SVM-linear-DGI-Chem	3.574	16.973	8.614	19.237	23.616	-2.345	0.625	0.625	-6.45	-4.757	2.736	-4.757	3.537	-9.859	-7.835	-12.244	7.633	7.234	19.251	-0.961	-6.029	-	0	0	0.001
VKR-DGI	-10.657	0.622	-6.064	5.128	-0.413	-17.959	-11.118	-11.118	-15.475	-19.575	-11.857	-19.575	-11.198	-35.319	-21.416	-27.315	-13.891	-10.212	-5.639	-20.455	-19.886	-16.32	-	0.008	0.373
VKR-Chem	-4.268	4.869	-1.71	6.351	13.391	-10.145	-8.087	-8.087	-15.248	-10.968	-4.361	-10.968	-3.692	-17.652	-20.123	-28.57	-4.825	-3.188	0.351	-10.472	-46.661	-21.272	4.871	-	0.208
VKR-DGI+Chem	-2.899	1.165	-1.655	1.726	0.88	-5.539	-4.414	-4.414	-9.223	-7.122	-2.998	-7.122	-2.664	-9.941	-8.736	-10.933	-2.936	-2.226	-1.47	-6.65	-8.105	-8.683	1.003	-1.499	-

We did a pairwise testing (paired t-test) on AUPR, giving evidence of whether the performance of methods outperforms the others or not. The upper triangular matrix in this matrix is the P-values of the paired t-test, and the lower triangular matrix is the t-statistics. We can see there are ‘nan’s in LNSM-CMI (both RLN and Jaccard) versus LNSM-Chem. The ‘nan’ here means the two results of nested CV are the same, as LNSM-CMI put more weight on the features with low loss function in the linear combination. Here we only use two features, leading to a zero coefficient for feature DGI, so the integrated feature is the same as the Chem.

Supplementary 3

Table S 3 P-value of AUROC

	Naive	KR-DGI	KR-Chem	MKR	KR-DGI+Chem	LNSM-RLN-DGI	LNSM-RLN-Chem	LNSM-CMI-RLN	LNSM-SMI-RLN	LNSM-Jaccard-DGI	LNSM-Jaccard-Chem	LNSM-CMI-Jaccard	LNSM-SMI-Jaccard	SVM-WGTS-DGI	SVM-WGTS-Chem	SVM-WGTS-DGI-Chem	SVM-RBF-DGI	SVM-RBF-Chem	SVM-RBF-DGI+Chem	SVM-linear-DGI	SVM-linear-Chem	SVM-linear-DGI-Chem	VKR-DGI	VKR-Chem	VKR-DGI+Chem
Naive	-	0	0.051	0.02	0.163	0.002	0.001	0.001	0	0.001	0.607	0.001	0.049	0	0	0	0	0.08	0.086	0	0	0	0	0.536	0.214
KR-DGI	11.089	-	0.683	0.66	0.687	0.016	0.002	0.002	0	0.062	0	0.062	0	0	0	0	0	0.016	0.062	0	0	0	0	0.003	0
KR-Chem	2.766	0.44	-	0.92	0.377	0.063	0.029	0.029	0	0.94	0.041	0.94	0.025	0	0.001	0.001	0.001	0.076	0.246	0	0	0	0.015	0.015	0.012
MKR	3.725	0.474	-0.106	-	0.463	0.009	0.001	0.001	0	0.793	0.023	0.793	0.012	0	0	0.001	0	0.039	0.043	0	0	0	0.004	0.005	0.003
KR-DGI+Chem	1.708	-0.433	-0.994	-0.81	-	0.019	0.005	0.005	0	0.488	0.15	0.488	0.086	0	0	0	0.001	0.28	0.527	0	0	0	0.048	0.02	0.066
LNSM-RLN-DGI	7.23	4.022	2.559	4.726	3.802	-	0.507	0.507	0	0.038	0.002	0.038	0.001	0	0.001	0.001	0.008	0.003	0.008	0.001	0	0.002	0.001	0.001	0.002
LNSM-RLN-Chem	8.903	6.882	3.342	9.46	5.727	0.727	-	nan	0	0.007	0.001	0.007	0.001	0	0	0.001	0.004	0	0	0	0	0	0	0	0
LNSM-CMI-RLN	8.903	6.882	3.342	9.46	5.727	0.727	nan	-	0	0.007	0.001	0.007	0.001	0	0	0.001	0.004	0	0	0	0	0	0	0	0
LNSM-SMI-RLN	23.771	26.651	19.646	27.825	20.543	13.598	28.18	28.18	-	0	0	0	0	0.001	0.104	0.377	0	0	0	0	0.003	0	0	0	0
LNSM-Jaccard-DGI	10.236	2.571	0.081	0.281	0.763	-3.049	-5.13	-5.13	-25.794	-	0.001	nan	0.001	0	0	0	0	0.024	0.036	0	0	0	0	0.007	0
LNSM-Jaccard-Chem	0.557	-10.461	-2.976	-3.587	-1.781	-7.239	-8.848	-8.848	-23.701	-8.303	-	0.001	0.001	0	0	0	0	0.061	0.104	0	0	0	0.001	0.41	0.172
LNSM-CMI-Jaccard	10.236	2.571	0.081	0.281	0.763	-3.049	-5.13	-5.13	-25.794	nan	8.303	-	0.001	0	0	0	0	0.024	0.036	0	0	0	0	0.007	0
LNSM-SMI-Jaccard	-2.802	-12.178	-3.497	-4.367	-2.267	-8.184	-9.769	-9.769	-24.158	-9.049	-10.127	-9.049	-	0	0	0	0	0.016	0.044	0	0	0	0.007	0.968	0.58
SVM-WGTS-DGI	46.456	49.582	26.385	46.332	21.968	26.638	29.833	29.833	8.236	57.233	42.937	57.233	43.389	-	0.004	0.014	0	0	0	0	0.001	0	0	0	0
SVM-WGTS-Chem	15.585	13.91	9.814	12.768	13.66	9.704	13.442	13.442	-2.095	12.914	15.512	12.914	15.872	-5.765	-	0.195	0.003	0	0	0.043	0.073	0.019	0	0	0
SVM-WGTS-DGI-Chem	13.041	11.419	8.645	9.756	10.489	7.947	9.494	9.494	-0.992	10.922	12.988	10.922	13.162	-4.156	1.557	-	0.005	0	0	0.044	0.086	0.03	0	0	0
SVM-RBF-DGI	15.672	16.568	9.972	14.129	7.942	4.837	6.032	6.032	-21.608	15.963	15.53	15.963	16.206	-54.065	-6.309	-5.519	-	0	0	0	0.002	0.005	0	0	0
SVM-RBF-Chem	2.329	-3.991	-2.384	-3.022	-1.249	-6.173	-11.358	-11.358	-28.644	-3.523	2.584	-3.523	4.002	-38.715	-15.382	-12.046	-16.372	-	0.627	0	0	0	0.006	0	0.011
SVM-RBF-DGI+Chem	2.27	-2.577	-1.358	-2.918	-0.692	-4.846	-22.237	-22.237	-37.305	-3.11	2.099	-3.11	2.914	-40.975	-17.892	-12.912	-14.816	0.525	-	0	0	0	0.01	0.025	0.022
SVM-linear-DGI	18.497	20.131	22.775	16.21	14.101	8.347	11.668	11.668	-13.538	18.731	19.286	18.731	19.739	-19.815	-2.937	-2.9	13.852	22.834	18.847	-	0.103	0.138	0	0	0
SVM-linear-Chem	16.252	15.921	16.469	18.571	59.632	11.472	18.528	18.528	-6.628	13.425	17.164	13.425	18.021	-9.976	-2.411	-2.267	7.256	24.233	20.973	2.105	-	0.006	0	0	0
SVM-linear-DGI-Chem	12.424	12.393	14.001	16.759	17.99	7.656	11.745	11.745	-14.526	10.917	12.802	10.917	13.415	-15.503	-3.78	-3.314	5.637	16.927	15.8	-1.847	-5.413	-	0	0	0
VKR-DGI	-10.9	-21.731	-4.125	-6.065	-2.816	-9.147	-11.161	-11.161	-26.19	-16.523	-7.912	-16.523	-5.038	-54.641	-15.824	-13.089	-19.968	-5.395	-4.591	-21.272	-17.745	-14.232	-	0.176	0.164
VKR-Chem	-0.677	-6.278	-4.105	-5.723	-3.737	-8.657	-14.972	-14.972	-28.312	-5.199	-0.918	-5.199	0.043	-36.634	-16.888	-12.895	-16.085	-11.151	-3.501	-21.832	-29.135	-18.66	1.643	-	0.615
VKR-DGI+Chem	-1.475	-11.736	-4.383	-6.498	-2.515	-7.717	-11.186	-11.186	-31.877	-10.899	-1.66	-10.899	-0.602	-70.019	-14.019	-11.539	-37.886	-4.476	-3.626	-30.155	-18.904	-17.557	1.704	-0.544	-

We also did the pairwise test (paired t-test) on AUROC to show evidence of whether methods significantly reach a higher value. The upper triangular matrix in this matrix is the P-values of the paired t-test, and the lower triangular matrix is the t-statistics. The reason nan in this matrix happens is the same as supplementary 2.

Supplementary 4

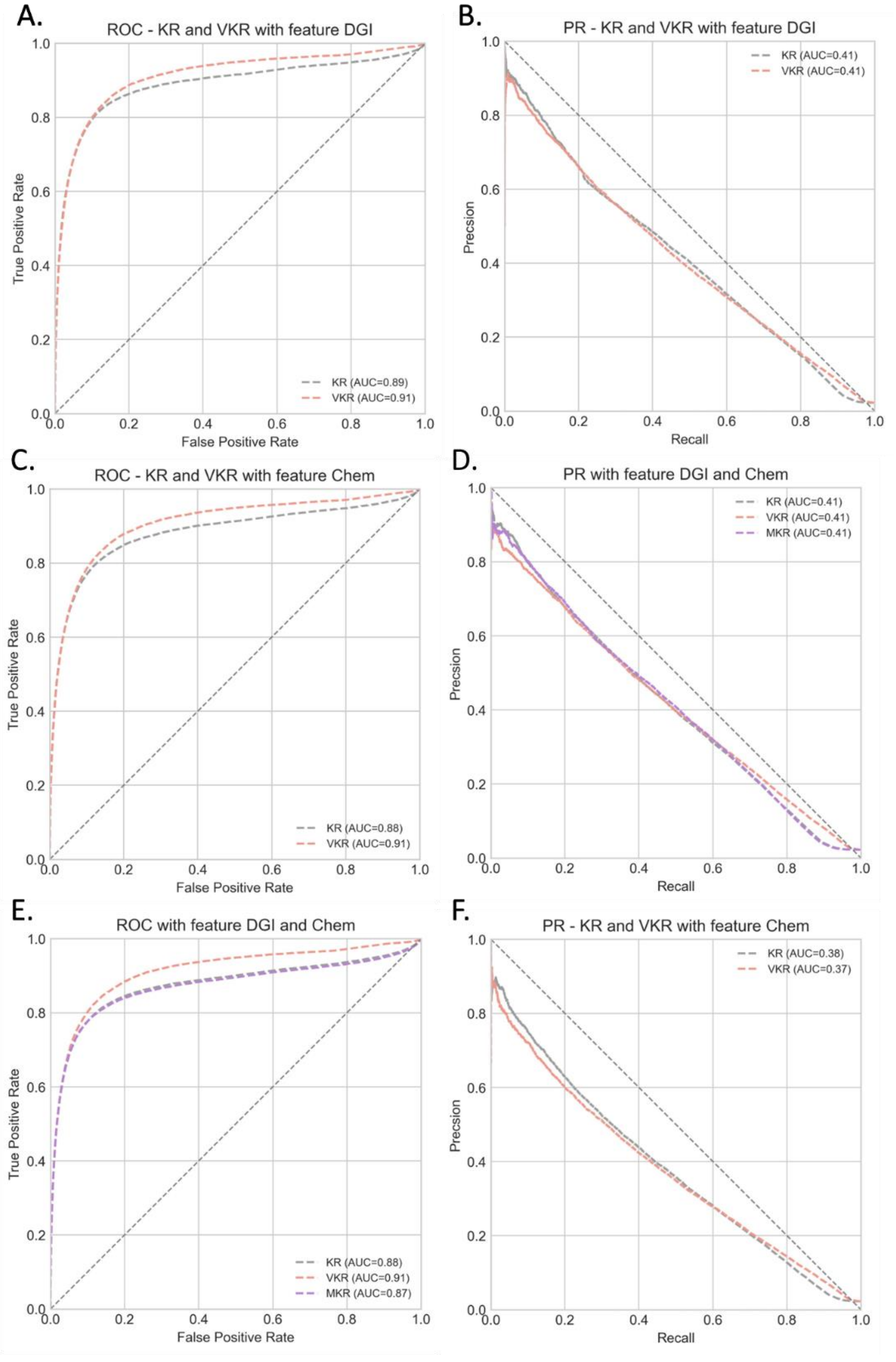

Figure S 1 Comparison of KR and VKR

A-B. The comparison between KR and VKR is based on DGI. VKR shows an improvement in AUROC, with AUPR at the same level. **C-D.** The comparison of KR and VKR. Using Chem shows a similar rule as DGI. **E-F.** The comparison of KR and VKR is based on the integration of DGI and Chem. An improvement in ROC is also shown in these subfigures.

Supplementary 5

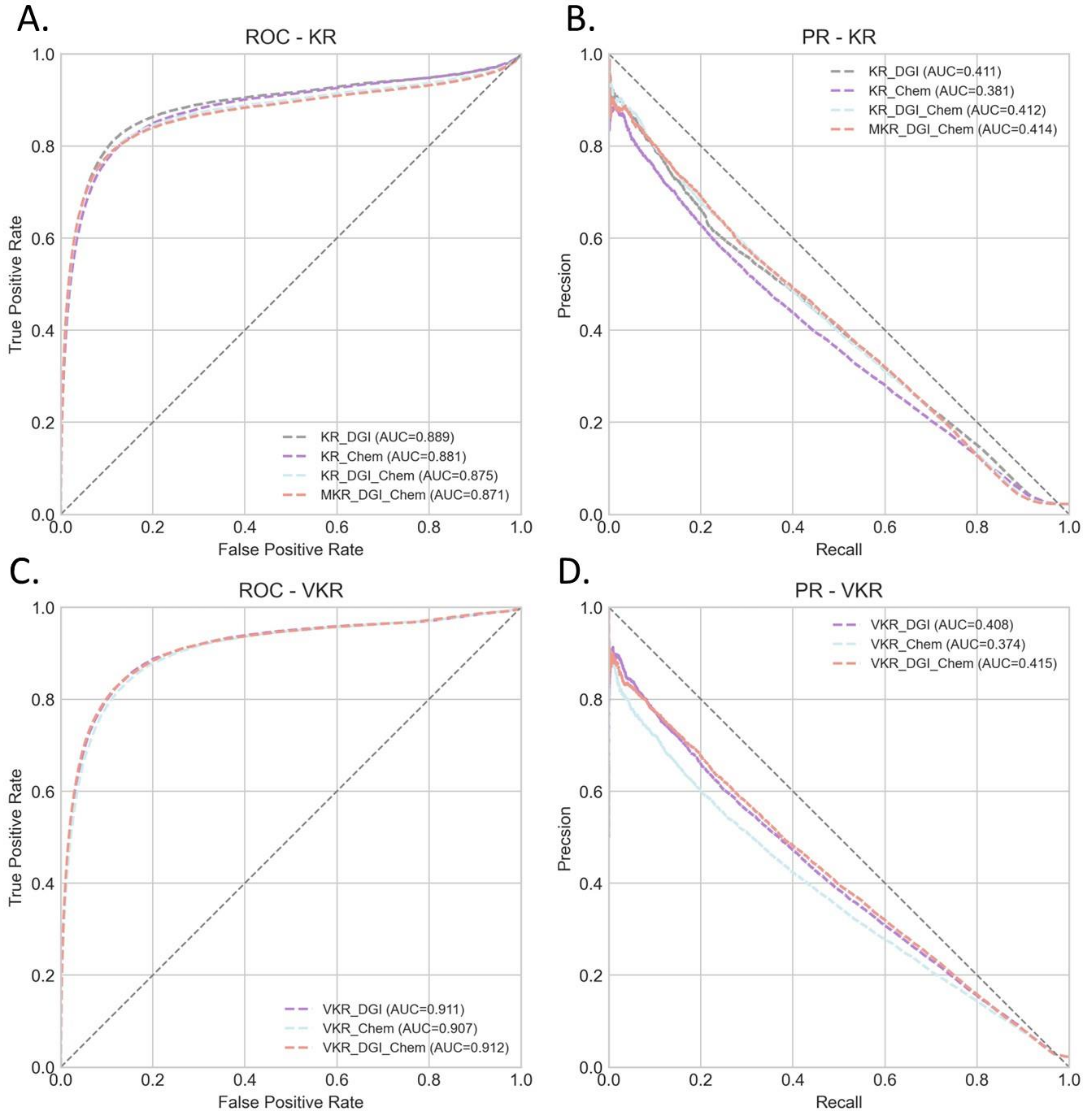

Figure S 2 Comparison of Features

A-B. The comparison between features using KR. **A.** The AUROC of KR based on DGI is the highest, followed by Chem and the integrations. **B.** The integration of features-based KR reaches the highest in AUPR, followed by DGI-based and then Chem-based. **C-D.** The comparison between features using VKR. **C.** The AUROC of VKR based on different features does not vary a lot. **D.** AUPR of integration of features is based on VKR rank the first, with a small advance better than DGI-based VKR. However, VKR with Chem shows worse performance in AUPR, like KR with Chem only.

Supplementary 6

Table S 4 The Summary of Existing Methods

Study	Method	Methods used to compare	Features	Cross Validation
GESSE: Predicting Drug Side Effects from Drug–Target Relationships (Pérez-Nueno *et al.*, 2015)	Ordinary Canonical Correlation Analysis (OCCA), Sparse CCA (SCCA), Regularized CCA (RCCA) + Discriminate Analysis (DA), Linear DA, Partial Least-Squares-DA	nan	Drug-target feature, compared with PubChem chemical substructure fingerprint	Leave-one-out (LOO) cross-validation (CV), 5-fold CV
Prediction of Adverse Drug Reactions Using Decision Tree Modeling (Hammann *et al.*, 2010)	Decision Tree (DT): (chi-squared automatic interaction detector) CHAID DT, classification and regression tree (CART)	nan	Structure-activity relationship	K-fold CV
Analysis of Pharmacology Data and the Prediction of Adverse Drug Reactions and Off Target Effects from Chemical Structure (Bender *et al.*, 2007)	Bayesian model	nan	Chemical-Structure	nan
Predicting Adverse Drug Reactions Using Publicly Available PubChem BioAssay Data (Pouliot *et al.*, 2011)	Logistic Regression (LR)	nan	Chemical-structure	LOOCV
Adverse Drug Reaction Prediction Using Scores Produced by Large-Scale Drug-Protein Target Docking on High-Performance Computing Machines (LaBute *et al.*, 2014)	L1 regularized LR	nan	Drug-protein target	10-fold CV
Drug-induced adverse events prediction with the LINCS L1000 data (Lu *et al.*, 2021)	Extra Tree (ET)	Random Forest (RF), Support vector Machine (SVM), LR, Naïve Bayes (NB), k-nearest neighbour (KNN)	L1000 Gene Expression, Chemical Structure, GO term enrichment	K-fold CV
Relating drug–protein interaction network with drug side effects (Mizutani *et al.*, 2012)	OCCA, SCCA	nan	Chemical structure, drug-protein interaction, drug-protein target	5-fold CV
Predicting drug side-effect profiles: a chemical fragment-based approach (Pauwels *et al.*, 2011)	OCCA, SCCA	KNN, SVM-RBF	Chemical substructure	5-fold CV
A unified frame of predicting side effects of drugs by using linear neighborhood similarity (Zhang *et al.*, 2017)	Linear Neighborhood Similarity Method, (LNSM), LNSM-Similarity Matrix Integration (SMI)	OCCA, SCCA	Chemical substructure, drug target, transporter, enzyme, pathway, treatment	5-fold CV
Drug Side-Effect Prediction Based on the Integration of Chemical and Biological Spaces (Yamanishi *et al.*, 2012)	Kernel Regression (KR), Multi-kernel Regression (MKR)	OCCA	Chemical substructure, drug-protein interaction	5-fold CV
Large-scale prediction of adverse drug reactions	SVM-RBF	LR, NB, KNN, RF	Chemical substructures,	5-fold CV

using chemical, biological, and phenotypic properties of drugs (Liu *et al.*, 2012)			drug target, transporter, enzymes, pathway	
Predicting adverse side effects of drugs (Huang *et al.*, 2011)	SVM-RBF, LR	nan	Human Annotated and Predicted Protein Interaction (HAPPI), Gene Ontology (GO)	10-fold CV
Drug Side-Effect Profiles Prediction: From Empirical to Structural Risk Minimization (Jiang *et al.*, 2020)	SVM-Weighted GTS, SVM-GTS	DT, KNN, SVM-RBF, SVM-linear, SVM-polynomial, Naïve Regression (NR), Regularized Regression (RR), LNSM	Chemical structure	5-fold CV
Predicting adverse drug reaction profiles by integrating protein interaction networks with drug structures (Huang *et al.*, 2013)	SVM-RBF	nan	HAPPI, chemical structure	10-fold CV
Metabolic Network Prediction of Drug Side Effects (Shaked, 2016)	SVM-linear, CCA (With AMPP)	CCA (With chemical structure)	Array of model based phenotype predictors (AMPP), chemical structure	LOOCV
Drug side effect prediction through linear neighborhoods and multiple data source integration (Zhang *et al.*, 2016)	LNSM, LNSM-SMI, LNSM-Cost Minimization Integration (CMI)	OCCA, SCCA, SVM-RBF, FS-MLKNN	Chemical substructure, drug target, transporter, enzyme, pathway, treatment	5-fold CV
Prediction of Side Effects Using Comprehensive Similarity Measures (Seo *et al.*, 2020)	RF, stacking of RF, LR, NB, XGBoost	RF	Drug-drug interaction, Single nucleotide polymorphism (SNP), target protein, chemical structure, indication	10-fold CV
Predicting Drug Side Effects Using Data Analytics and the Integration of Multiple Data Sources (Lee *et al.*, 2017)	NB, KNN, RF in three intervals, NB, KNN, RF	SVM, OCCA, SCCA	chemical substructure, protein, indications	10-fold CV
MEDICASCY: A Machine Learning Approach for Predicting Small-Molecule Drug Side Effects, Indications, Efficacy, and Modes of Action (Zhou *et al.*, 2020)	Boosted RF	LNSM, FSMLKNN, multi-layer perceptron, KNN, KG-SIM-PROP, K-means, RF	Chemical structure (fingerprint OpenBabel FP2 FP3, FP4, MACCS), drug-protein target, drug-disease-associated protein	LOOCV

The summary of the existing methods. The existing methods might consider both ADR prediction for new drugs and potential ADRs for known drugs. Here we only consider the ADR prediction for new drugs. Therefore, those methods for known drugs are not in column 'Methods used to compare'.

Supplementary 7

Table S 5 AUPR of Nested CV Each Fold

Method and features	Fold 1	Fold 2	Fold 3	Fold 4	Fold 5
Naïve	0.3755	0.3754	0.3599	0.3749	0.3718
KR-DGI	0.4181	0.4331	0.4339	0.4439	0.4449
KR-Chem	0.4030	0.3848	0.3836	0.4104	0.3968
MKR	0.4273	0.4383	0.4425	0.4493	0.4530
KR-DGI+Chem	0.4331	0.4122	0.4279	0.4409	0.4432
LNSM-RLN-DGI	0.3257	0.3139	0.3225	0.3078	0.3209
LNSM-RLN-Chem	0.3574	0.3457	0.3260	0.3550	0.3467
LNSM-CMI-RLN	0.3574	0.3457	0.3260	0.3550	0.3467
LNSM-SMI-RLN	0.2945	0.2707	0.2937	0.2880	0.3276
LNSM-Jaccard-DGI	0.2877	0.2986	0.2852	0.2730	0.3009
LNSM-Jaccard-Chem	0.3700	0.3696	0.3645	0.3600	0.3676
LNSM-CMI-Jaccard	0.2877	0.2986	0.2852	0.2730	0.3009
LNSM-SMI-Jaccard	0.3763	0.3756	0.3710	0.3680	0.3739
SVM-WGTS-DGI	0.2414	0.2503	0.2429	0.2531	0.2313
SVM-WGTS-Chem	0.2852	0.2674	0.2581	0.2846	0.2679
SVM-WGTS-DGI-Chem	0.2585	0.2388	0.2438	0.2714	0.2443
SVM-RBF-DGI	0.3768	0.3727	0.3749	0.3834	0.3931
SVM-RBF-Chem	0.3889	0.3817	0.3787	0.3983	0.3875
SVM-RBF-DGI+Chem	0.3985	0.3913	0.3911	0.4220	0.4166
SVM-linear-DGI	0.3230	0.3210	0.3420	0.3344	0.3564
SVM-linear-Chem	0.3191	0.2967	0.3062	0.3260	0.3267
SVM-linear-DGI-Chem	0.3297	0.3187	0.3361	0.3595	0.3588
VKR-DGI	0.4201	0.4290	0.4377	0.4427	0.4386
VKR-Chem	0.4034	0.3802	0.3950	0.4199	0.4160
VKR-DGI+Chem	0.3846	0.3900	0.4059	0.4601	0.4599

AUPR of 5-fold nested CV. We set a random seed for nested CV, which means we ran every method on the same subset of data.

Supplementary 8

Table S 6 AUROC of Nested CV Each Fold

Method and features	Fold 1	Fold 2	Fold 3	Fold 4	Fold 5
Naïve	0.9070	0.9086	0.9106	0.9091	0.9059
KR-DGI	0.8889	0.8912	0.8927	0.8939	0.8954
KR-Chem	0.8781	0.8736	0.9060	0.8866	0.9047
MKR	0.8825	0.8819	0.8865	0.9068	0.8950
KR-DGI+Chem	0.8983	0.8668	0.9005	0.9039	0.9075
LNSM-RLN-DGI	0.8656	0.8573	0.8774	0.8881	0.8592
LNSM-RLN-Chem	0.8659	0.8546	0.8561	0.8764	0.8731
LNSM-CMI-RLN	0.8659	0.8546	0.8561	0.8764	0.8731
LNSM-SMI-RLN	0.7297	0.7324	0.7234	0.7425	0.7610
LNSM-Jaccard-DGI	0.8838	0.8928	0.8879	0.8897	0.8921
LNSM-Jaccard-Chem	0.9070	0.9058	0.9120	0.9077	0.9066
LNSM-CMI-Jaccard	0.8838	0.8928	0.8879	0.8897	0.8921
LNSM-SMI-Jaccard	0.9099	0.9078	0.9150	0.9115	0.9094
SVM-WGTS-DGI	0.6679	0.6928	0.6837	0.6954	0.6898
SVM-WGTS-Chem	0.7906	0.7474	0.7372	0.7608	0.7610
SVM-WGTS-DGI-Chem	0.7954	0.7472	0.7299	0.7364	0.7487
SVM-RBF-DGI	0.8199	0.8344	0.8353	0.8407	0.8472
SVM-RBF-Chem	0.8997	0.8912	0.9036	0.9047	0.9070
SVM-RBF-DGI+Chem	0.9001	0.8945	0.8908	0.9067	0.9065
SVM-linear-DGI	0.7815	0.7897	0.7978	0.7934	0.8162
SVM-linear-Chem	0.7855	0.7538	0.7789	0.7901	0.7972
SVM-linear-DGI-Chem	0.7928	0.7843	0.8012	0.8176	0.8281
VKR-DGI	0.9124	0.9157	0.9183	0.9185	0.9148
VKR-Chem	0.9107	0.8979	0.9131	0.9159	0.9154
VKR-DGI+Chem	0.9027	0.9098	0.9146	0.9166	0.9179

AUROC of 5-fold nested CV. We set a random seed for nested CV, which means we ran every method on the same subset of data.

Supplementary 9

Table S 7 Hyperparameters of All Methods for Nested CV and Hold-out Set

Method and features	Fold 1	Fold 2	Fold 3	Fold 4	Fold5	test set
Naïve						
KR-DGI	$\lambda = 0.1, \sigma = 10$	$\lambda = 0.1, \sigma = 10$	$\lambda = 0.1, \sigma = 10$	$\lambda = 0.1, \sigma = 10$	$\lambda = 1, \sigma = 10$	$\lambda = 0.1, \sigma = 10$
KR-Chem	$\lambda = 10, \sigma = 10$	$\lambda = 1, \sigma = 10$	$\lambda = 1, \sigma = 10$	$\lambda = 10, \sigma = 10$	$\lambda = 10, \sigma = 10$	$\lambda = 1, \sigma = 10$
MKR	$\lambda_1 = 0.1, \lambda_2 = 1000, \sigma = 10$	$\lambda_1 = 0.1, \lambda_2 = 100, \sigma = 10$	$\lambda_1 = 1, \lambda_2 = 10, \sigma = 10$	$\lambda_1 = 0.1, \lambda_2 = 100, \sigma = 10$	$\lambda_1 = 1, \lambda_2 = 100, \sigma = 10$	$\lambda_1 = 0.1, \lambda_2 = 1, \sigma = 10$
KR-DGI+Chem	$\lambda = 1, \sigma = 10$	$\lambda = 0.1, \sigma = 10$	$\lambda = 1, \sigma = 10$	$\lambda = 1, \sigma = 10$	$\lambda = 1, \sigma = 10$	$\lambda = 0.1, \sigma = 10$
LNSM-RLN-DGI	$\alpha = 0.1$	$\alpha = 0.1$	$\alpha = 0.1$	$\alpha = 0.1$	$\alpha = 0.1$	$\alpha = 0.1$
LNSM-RLN-Chem	$\alpha = 0.25$	$\alpha = 0.25$	$\alpha = 0.3$	$\alpha = 0.3$	$\alpha = 0.3$	$\alpha = 0.15$
LNSM-CMI-RLN	$\alpha = 0.25$	$\alpha = 0.25$	$\alpha = 0.3$	$\alpha = 0.3$	$\alpha = 0.3$	$\alpha = 0.15$
LNSM-SMI-RLN	$\alpha = 0.1$	$\alpha = 0.1$	$\alpha = 0.1$	$\alpha = 0.1$	$\alpha = 0.1$	$\alpha = 0.1$
LNSM-Jaccard-DGI	$\alpha = 0.1$	$\alpha = 0.1$	$\alpha = 0.1$	$\alpha = 0.1$	$\alpha = 0.1$	$\alpha = 0.1$
LNSM-Jaccard-Chem	$\alpha = 0.1$	$\alpha = 0.1$	$\alpha = 0.1$	$\alpha = 0.1$	$\alpha = 0.1$	$\alpha = 0.1$
LNSM-CMI-Jaccard	$\alpha = 0.1$	$\alpha = 0.1$	$\alpha = 0.1$	$\alpha = 0.1$	$\alpha = 0.1$	$\alpha = 0.1$
LNSM-SMI-Jaccard	$\alpha = 0.1$	$\alpha = 0.1$	$\alpha = 0.1$	$\alpha = 0.1$	$\alpha = 0.1$	$\alpha = 0.1$
SVM-WGTS-DGI	$C = 10^{-7}$	$C = 10^{-7}$	$C = 10^{-8}$	$C = 10^{-8}$	$C = 10^{-8}$	$C = 10^{-7}$
SVM-WGTS-Chem	$C = 10^{-7}$	$C = 10^{-8}$	$C = 10^{-7}$	$C = 10^{-5}$	$C = 10^{-7}$	$C = 10^{-5}$
SVM-WGTS-DGI-Chem	$C = 10^{-8}$	$C = 10^{-8}$	$C = 10^{-7}$	$C = 10^{-8}$	$C = 10^{-8}$	$C = 10^{-5}$
SVM-RBF-DGI	$C = 0.1, \sigma = 0.0001$	$C = 0.1, \sigma = 0.0001$	$C = 0.1, \sigma = 0.0001$	$C = 0.1, \sigma = 0.0001$	$C = 0.1, \sigma = 0.0001$	$C = 0.1, \sigma = 0.001$
SVM-RBF-Chem	$C = 0.1, \sigma = 0.01$	$C = 0.1, \sigma = 0.01$	$C = 0.1, \sigma = 0.01$	$C = 0.1, \sigma = 0.01$	$C = 0.1, \sigma = 0.01$	$C = 0.1, \sigma = 0.01$
SVM-RBF-DGI+Chem	$C = 0.1, \sigma = 0.001$	$C = 0.1, \sigma = 0.001$	$C = 0.1, \sigma = 0.001$	$C = 0.1, \sigma = 0.001$	$C = 0.1, \sigma = 0.001$	$C = 0.1, \sigma = 0.001$
SVM-linear-DGI	$C = 0.001$	$C = 0.001$	$C = 0.001$	$C = 0.001$	$C = 0.001$	$C = 0.001$
SVM-linear-Chem	$C = 0.001$	$C = 0.001$	$C = 0.001$	$C = 0.001$	$C = 0.001$	$C = 0.001$
SVM-linear-DGI-Chem	$C = 0.001$	$C = 0.001$	$C = 0.001$	$C = 0.001$	$C = 0.001$	$C = 0.001$
VKR-DGI	$\lambda = 0.1, \sigma = 10, k = 20$	$\lambda = 0.1, \sigma = 10, k = 20$	$\lambda = 0.1, \sigma = 10, k = 20$	$\lambda = 0.1, \sigma = 10, k = 10$	$\lambda = 0.1, \sigma = 10, k = 15$	$\lambda = 0.01, \sigma = 10, k = 20$
VKR-Chem	$\lambda = 0.01, \sigma = 100, k = 10$	$\lambda = 0.1, \sigma = 10, k = 20$	$\lambda = 0.001, \sigma = 100, k = 20$	$\lambda = 0.001, \sigma = 100, k = 10$	$\lambda = 0.01, \sigma = 100, k = 15$	$\lambda = 1, \sigma = 10, k = 25$
VKR-DGI+Chem	$\lambda = 0.1, \sigma = 10, k = 30$	$\lambda = 0.01, \sigma = 10, k = 20$	$\lambda = 0.1, \sigma = 10, k = 30$	$\lambda = 0.1, \sigma = 10, k = 20$	$\lambda = 0.1, \sigma = 10, k = 30$	$\lambda = 0.01, \sigma = 10, k = 25$

For the hyperparameters in the LNSM family, α was tuned with the strategy from 0.5 to 1 with step 0.5. And for the num of component k in VKR, we tune it starting with 5 and step 5. The rest of the tunings follow the strategy of $\{\ldots, 10^{-2}, 10^{-1}, 10^{0}, 10^{1}, 10^{2}, \ldots\}$.

Supplementary 10

A Brief Review of KR:

KR was first used in (Yamanishi *et al.*, 2012) ADR prediction study. It turns out to be Kernel Ridge Regression. The objective function of KR is shown as follows.

$$\left\|Y^{\mathrm{T}} - KW\right\|^2 + \lambda\|W\|^2, \quad \text{(S 1)}$$

where $Y = [y_1, y_2, \dots, y_i, \dots, y_N]$ is the drug-ADR matrix (Yamanishi *et al.*, 2012), and $y_i$is the ADRs vector of drug i, K is a kernel matrix, K is a coefficient matrix. By minimizing the objective function, W can be fiited. λ is a regularization parameter. Gaussian radial basis function (RBF) kernel was used in this study. Therefore, entries in K were defined as an inner product $k_{i,j} = k(x_i, x_j) = \exp\left(\frac{\|x_i - x_j\|^2}{2\sigma^2}\right)$, where σ is the width parameter, and $x_i$is the feature vector of drug i.

For ADR prediction, the analytical solution of W can be obtained by solving the equation:

$$(K^2 + \lambda I)W = KY^{\mathrm{T}}, \quad \text{(S 2)}$$

and the prediction of Y can be calculated by the following equation:

$$Y^{\mathrm{new\,T}} = K^{\mathrm{new}}Y^{\mathrm{T}}, \quad \text{(S 3)}$$

where $Y^{\mathrm{new}} = [y_1^{\mathrm{new}}, y_2^{\mathrm{new}}, \dots, y_i^{\mathrm{new}}, \dots, y_{N'}^{\mathrm{new}}]$, y_i^{new} represents the ADRs of new drug i, and K^{new} is defined as $k_{i,j}^{\mathrm{new}} = k(x_i^{\mathrm{new}}, x_j) = \exp\left(\frac{\|x_i^{\mathrm{new}} - x_j\|^2}{2\sigma^2}\right)$.

The ridge term in Equation S 1 can reduce the variance, but causes an increase in bias, and finally increases the error of the prediction, and high bias is in some way due to the noise of data. To reduce the error, we can de-noise the drug-ADR data by a dimension reduction method NMF.

For the integration of features, two methods were carried out. One is the linear combination of the kernel matrix. the objective function is as follows:

$$\left\|Y^{\mathrm{T}} - (\sum_l K_l)W\right\|^2 + \lambda\|W\|^2, \quad \text{(S 4)}$$

where l is the lth feature. ($l = 2$ in this study representing DGI and Chem respectively)

and the other is Multi-kernel Regression (MKR):

$$\left\|Y^{\mathrm{T}} - \sum_l K_l W_l\right\|^2 + \sum_l \lambda_l\|W_l\|^2, \quad \text{(S 5)}$$

which introduces more hyperparameters than the linear combination of kernel (λ_1, λ_2 for DGI and Chem compared with λ in equation S 4).

Supplementary 11

A Brief Review of NMF:

NMF is one of the dimension reduction methods that can de-noise drugs or ADRs into K components. It is a method that analyzes the non-negative matrix by finding $U = [u_{il}] \in \mathbb{R}^{M \times L}$ and $V = [v_{jl}] \in \mathbb{R}^{N \times L}$. Here U, V are two non-negative matrices, with components L. By the following equation, they can approximate the round truth matrix Y (Lee and Seung, 1999):

$$Y \approx UV^{\mathrm{T}}. \tag{S 6}$$

One of the common objective functions of NMF, qualifying the quality of the approximation, is the *Frobenius norm* (Paatero and Tapper, 1994):

$$\|Y - UV^{T}\|^{2}\ , \tag{S 7}$$

The objective function is convex in U only and V only, which means it is not convex in both simultaneously. Therefore, only a local minimum can be found for Equation S 5. (Lee and Seung, 2000) provide updating rules for U and V as follows:

$$u_{il} \leftarrow u_{il} \frac{(YU)_{il}}{(UV^{\mathrm{T}}V)_{il}}, v_{jl} \leftarrow \frac{\left(Y^{\mathrm{T}}U\right)_{jl}}{(VU^{\mathrm{T}}U)_{jl}}, \tag{S 8}$$

where $U = [u_{il}], V = [v_{jl}]$, and $(A)_{il}$ is the (i, l) entry of matrix A. Notice that NMF cannot directly solve the ADR prediction problem since columns of Y are all zeros. This results in the rows of new drugs in V remaining 0 and further leads to the zeros prediction of Y^{new} (Figure 1 A); despite that NMF is a suitable method to reduce the number of ADRs in Y to a small number of generated features in V.

Supplementary 12

We reviewed some representative methods that were filtered by specific rules. Three rules were designed to choose methods, in order to cover most of the existing methods. We also compared VKR and the Naïve method with these existing methods. We summarized part of the advanced method worth studying and gave an overview of how methods perform in corresponding studies (Table 2). The rules of offering methods for review and comparison are stated as follows.

We considered the advanced methods. SVM was found as the most commonly used method, so we filtered those that outperformed SVM. (Liu *et al.*, 2012) compared SVM-RBF with other classical methods, including LR, NB, KNN, and RF, and SVM-RBF performs the best in ADR predictions. In addition, SVM-RBF was also used to compare with other advanced methods and outperformed them (Zhang *et al.*, 2016). However, some studies did not compare with SVM such as KR (Yamanishi *et al.*, 2012), In total, there are 3 types of methods: methods outperform SVM (LNSM family, SVM-WGTS), methods did not compare with SVM (KR, MKR, MEDICASCY, (Seo *et al.*, 2020), the Naïve method), and methods underperform (or equivalent to) SVM. By comparing the first two types, we can have a brief understanding of how VKR performs compared with most of the existing methods.

Removing additional strategies on data. Additionally, there are many subtypes of methods in ADR prediction studies. Researchers improved the performance of methods using different strategies on data, for example, feature selection or other strategies. Huang *et al.* (2013) and Huang *et al.* (2011) used feature selection before the classifier, but SVM was used as their method. Lee *et al.* (2017) divided drug-ADR data according to the frequency of ADR into three intervals. We did not consider this study comparable, because it is essentially running existing methods on different subsets of data. Other methods might also be improved with such strategies. One of our aims is to study what existing methods can make a good prediction of ADRs. Hence, to make methods comparable, we should not consider additional strategies for data.

If the methods have equivalent performance, we only considered one of them. For those methods that have equivalent results compared with others, we only considered one of them to reduce time and computational resource consumption. Seo *et al.* (2020) stacked 4 methods together, and it turns out that the proposed method returned a close result as RF. The method used in MEDICASCY (Boosted RF, BRF) was surpassed by LNSM-SMI in some versions. Even the top version of MEDICASCY can only reach a slight improvement in the performance of LNSM-SMI (Zhou *et al.*, 2020), so we considered BRF to be approximately equivalent to LNSM-SMI. By considering some methods have equivalent prediction performance to others, we can reduce the complexity of comparison. Since RF and LNSM-SMI are also frequently shown in other studies, we deem that RF and LNSM-SMI can be the equivalent methods (Seo *et al.*, 2020, Zhou *et al.*, 2020). As RF was found to outrun by SVM-RBF, SVM-RBF happens to be a better option to review and compare in this study.

By considering all three rules for filtering methods, LNSM, LNSM-CMI, LNSM-SMI, KR, MKR, and SVM with kernel RBF and WGTS (highlighted methods shown in Table 2) were selected to be studied. For all the methods we compared, we tried not to be aggressive in tuning hyperparameters. In most cases, we tuned methods according to the same strategies in VKR (detailed strategies can be seen in Supplementary 7). Moreover, we also referred to the hyperparameters tuning strategies in the corresponding study.

References:

Bender,A. et al. (2007) Analysis of Pharmacology Data and the Prediction of Adverse Drug Reactions and Off-Target Effects from Chemical Structure. ChemMedChem, 2, 861–873.

Bender,A. et al. (2007) Analysis of Pharmacology Data and the Prediction of Adverse Drug Reactions and Off-Target Effects from Chemical Structure. ChemMedChem, 2, 861–873.

Huang,L.-C. et al. (2011) Predicting adverse side effects of drugs. BMC Genomics, 12, S11.

Jiang,H. et al. (2020) Drug Side-Effect Profiles Prediction: From Empirical to Structural Risk Minimization. IEEE/ACM Transactions on Computational Biology and Bioinformatics, 17, 402–410.

LaBute,M.X. et al. (2014) Adverse Drug Reaction Prediction Using Scores Produced by Large-Scale Drug-Protein Target Docking on High-Performance Computing Machines. PLOS ONE, 9, e106298.

Lee,D. and Seung,H.S. (2000) Algorithms for Non-negative Matrix Factorization. In, Advances in Neural Information Processing Systems. MIT Press.

Lee,D.D. and Seung,H.S. (1999) Learning the parts of objects by non-negative matrix factorization. Nature, 401, 788–791.

Lee,W.-P. et al. (2017) Predicting Drug Side Effects Using Data Analytics and the Integration of Multiple Data Sources. IEEE Access, 5, 20449–20462.

Liu,M. et al. (2012) Large-scale prediction of adverse drug reactions using chemical, biological, and phenotypic properties of drugs. Journal of the American Medical Informatics Association, 19, e28–e35.

Lu,J. et al. (2021) Drug-induced cell viability prediction from LINCS-L1000 through WRFEN-XGBoost algorithm. BMC Bioinformatics, 22, 13.

Mizutani,S. et al. (2012) Relating drug-protein interaction network with drug side effects. Bioinformatics, 28, i522–i528.

Paatero,P. and Tapper,U. (1994) Positive matrix factorization: A non-negative factor model with optimal utilization of error estimates of data values. Environmetrics, 5, 111–126.

Pauwels,E. et al. (2011) Predicting drug side-effect profiles: a chemical fragment-based approach. BMC Bioinformatics, 12, 169.

Pérez-Nueno,V.I. et al. (2015) GESSE: Predicting Drug Side Effects from Drug–Target Relationships. J. Chem. Inf. Model., 55, 1804–1823.

Pouliot,Y. et al. (2011) Predicting Adverse Drug Reactions Using Publicly Available PubChem BioAssay Data. Clinical Pharmacology & Therapeutics, 90, 90–99.

Seo,S. et al. (2020) Prediction of Side Effects Using Comprehensive Similarity Measures. BioMed Research International, 2020, e1357630.

Shaked,I. (2016) Metabolic Network Prediction of Drug Side Effects. 6.

Yamanishi,Y. et al. (2012) Drug Side-Effect Prediction Based on the Integration of Chemical and Biological Spaces. J. Chem. Inf. Model., 52, 3284–3292.

Zhang,W. et al. (2017) A unified frame of predicting side effects of drugs by using linear neighborhood similarity. BMC Syst Biol, 11, 101.

Zhang,W. et al. (2016) Drug side effect prediction through linear neighborhoods and multiple data source integration. 8.

Zhou,H. et al. (2020) MEDICASCY: A Machine Learning Approach for Predicting Small-Molecule Drug Side Effects, Indications, Efficacy, and Modes of Action. Mol. Pharmaceutics, 17, 1558–1574.
